# Bensulfuron-Methyl Treatment of Soil Affects the Infestation of Whitefly, Aphid, and *Tobacco Mosaic Virus* on *Nicotiana tabacum*

**DOI:** 10.3389/fpls.2016.01970

**Published:** 2016-12-26

**Authors:** Renyi Li, Saif Ul Islam, Zujian Wu, Xiujuan Ye

**Affiliations:** ^1^Key Laboratory of Plant Virology of Fujian Province, Institute of Plant Virology, Fujian Agriculture and Forestry UniversityFuzhou, China; ^2^Key laboratory of Biopesticide and Chemical Biology, Ministry of Education, Fujian Agriculture and Forestry UniversityFuzhou, China

**Keywords:** herbicide, *Bemisia tabaci*, *Myzus persicae*, *Tobacco mosaic virus*, jasmonic acid, salicylic acid

## Abstract

Bensulfuron-methyl (BSM) is widely used in paddy soil for weed control. BSM residue in the soil has been known to inhibit the growth of sensitive crop plants. However, it is unknown whether BSM residue can affect the agrosystem in general. In this study, we have found significant effects of BSM on the infestation of *Bemisia tabaci*, *Myzus persicae*, and *Tobacco mosaic virus* (TMV) in *Nicotiana tabacum*. The soil was treated with BSM before the pest inoculation. The herbicide-treated tobaccos showed resistance to *B. tabaci*, but this resistance could not be detected until 15-day post-infestation when smaller number of adults *B. tabaci* appeared. In *M. persicae* assay, the longevity of all development stages of insects, and the fecundity of insects were not significantly affected when feeding on BSM-treated plants. In TMV assay, the BSM treatment also reduced virus-induced lesions in early infection time. However, the titer of TMV in BSM treated plants increased greatly over time and was over 40-fold higher than the mock-infected control plants after 20 days. Further studies showed that BSM treatment increased both jasmonic acid (JA) and salicylic acid (SA) levels in tobacco, as well as the expression of target genes in the JA and SA signaling pathways, such as *NtWIPK*, *NtPR1a*, and *NtPAL. NtPR1a* and *NtPAL* were initially suppressed after virus-inoculation, while *NtRDR1* and *NtRDR6*, which play a key role in fighting virus infection, only showed up- or were down-regulated 20 days post virus-inoculation. Taken together, our results suggested that BSM residue in the soil may affect the metabolism of important phytohormones such as JA and SA in sensitive plants and consequently affect the plant immune response against infections such as whitefly, aphids, and viruses.

## Introduction

Herbicides are nowadays widely used to control weeds in order to reduce the cost of labor (Powles, 2014). Properties such as low-toxicity, environmental-friendly, and high-selectivity of the herbicides are desirable (Busi et al., 2013). Bensulfuron-methyl (BSM), which was developed in 1970s, is a herbicide belonging to the sulfonylurea class (Saeki and Toyota, 2004) and used in paddy fields (Lin et al., 2012). The mechanism of BSM-mediated weed killing involves the inhibition of *acetolactate synthase* (ALS) and the biosynthesis of branched-chain amino acids (Saeki and Toyota, 2004). This herbicide shows high-selectivity and also appears harmless to the Poaceae crops such as rice and maize (Saika et al., 2014). However, over-utilization can have negative impact on farming by creating herbicide-resistant weeds (Powles and Yu, 2010; Walsh and Powles, 2014) and damaging sensitive crops such as tomatoes and tobaccos (Lin et al., 2012).

Both *Bemisia tabaci* (Gennadius) and *Myzus persicae* (Sulzer) are serious pests affecting the production of tobaccos and other crops (Kollenberg et al., 2014; Santos-Garcia et al., 2015; Cao et al., 2016; Kang et al., 2016). Also maize fields have been reported to be damaged by the colonization of biotype B whiteflies (Quintela et al., 2016). *B. tabaci* not only causes disintegration of chloroplasts, sooty mold by honeydew, and lower carotenoid in many plants, especially the ones belonging to Solanaceae (Masuda et al., 2016), but carries and transmits several species of Geminiviruses which can lower crop yields tremendously (Shi et al., 2014a). Owing to fast evolution, *B. tabaci* nowadays has diverse biotypes worldwide among which the B and Q types can cause severe crops losses (Santos-Garcia et al., 2015). Green peach aphid *M. persicae*, which is another kind of important pest affecting tobaccos, has a broad spectrum of hosts all over the world (Cao et al., 2016; Kang et al., 2016; Kayser et al., 2016). The pests can both deprive plants of nutrients and transmit cucumber mosaic viruses or other pathogens (Zhao et al., 2015), resulting in immense losses of crop yield (Cao et al., 2016). *Tobacco mosaic virus* (TMV) is widely spread around the world, and it can affect a broad spectrum of hosts such as tobaccos, tomatoes, and potatoes, leading to economical losses.

The interaction between plants and pests/pathogens is affected by various factors such as chemicals, light, salt, temperature, and also biological factors (Shavit et al., 2013; Shi et al., 2014b; Su et al., 2016). In this regard, a number of studies have shown that the interaction depends on the endogenous levels of jasmonic acid (JA) and salicylic acid (SA) in host plants. Shi et al. (2014b) investigated three signal pathways involving JA, SA, and proteinase inhibitor (PI) in the plant-whitefly interaction and discovered that non-viruliferous and viruliferous B-type can induce different defense pathways in host plants. In addition, the infestation of Chinese cabbage *Brassica* by aphids can strongly induce the production of SA, but exogenous application of SA doesn’t have any effect on the performance of aphids (Cao et al., 2016). It has been founded that the replication of TMV can be inhibited by the application of SA (Conti et al., 2012). In the interaction between viruses and phytohormones, *RNA-dependent RNA polymerase* (*RDR*) family is involved in the establishing of the resistance in host plants. In *MtRDR1* transgenic tobaccos, *MtRDR1* can function as *RDR6* and can help the host plants to recover from the symptom caused by virus infection in the presence of SA (Lee et al., 2016).

Jasmonic acid and SA accumulate in host plants via a complex network of response and biosynthesis pathways (Turner et al., 2002). *Wound induced protein kinase* (*WIPK*) plays a key role in the JA production induced by wound or biting of herbivores and can suppress SA accumulation (Turner et al., 2002). *Phospholipase D* (*PLD*), *lipoxygenase* (*LOX*), *allene oxide synthase* (*AOS*), *allene oxide cyclase* (*AOC*), *OPDA reductase* (*OPR*) are involved in the biosynthesis of JA from chloroplast membrane lipids in plants (Turner et al., 2002). In the biosynthesis of SA, *phenylalanine ammonia lyase* (*PAL*) catalyzes the first step of the biosynthesis of SA (Shah, 2003; Chen et al., 2009), and SA can also induce the expression of *pathogenesis-related gene* (*PR*; Turner et al., 2002; D’Maris et al., 2010). In addition, *isochorismate synthase* (*ICS*) that was discovered in bacterial has been shown to be the key enzyme in the biosynthesis pathway of SA (Shah, 2003; Chen et al., 2009).

So far, few studies have directly addressed the herbicide residue problem in contaminated soils and the effect on susceptible crops. In this study, we focus on the ecological effect of BSM on tobaccos and on the interactions between the plants and pests/viruses. We also investigate physiological changes involving JA and SA signaling pathway induced by the herbicide residue.

## Materials and Methods

### Plant Materials, Insects, and Viruses

Seedlings of *Nicotiana tabacum* K326 were germinated under the following conditions: 28°C, 16 h of light and 8 h of dark. We used 0.25 mg⋅kg^-1^ as the most appropriate concentration of BSM to mimic the herbicide residue in the soil (Dry Weight, DW; Saeki and Toyota, 2004). Ten days post chemical-treatment, the symptoms caused by the herbicide were clearly visible. Plants not treated with BSM were used as controls. Treated and untreated (control) plants were then used for different experiments, i.e., pests infestation, pests feeding, Virus-induced symptom-evaluation, TMV inoculation RNA extraction, RT-qPCR assay, and JA and SA contents determination.

Non-viruliferous *B. tabaci*, *M. persicae*, and TMV strains isolated from Fujian Province (China) were kept on different plants of *N. tabacum* in the laboratory to be used for the investigation.

### *B. tabaci* Infestation

In the experiments, plants were tested pairwise. One plant has been treated with BSM in the soil for 10 days, and the other was an untreated control. Each pair of the plants were infested with 50 adults of non-viruliferous whitefly, who could choose any plant to feed on. We chose to calculate the number of adults on the third to fifth leaves at 1, 3, 5, 10, 15, and 20 days post the infestation because the majority of adults chose to feed on those leaves. At the same time the size of corresponding leaves was measured in order to calculate the density of insects by the equation below:

S=0.6345ab

*S*, The size of the leaf; *a*, The length of the leave; *b*, The width of the leaf.

This leaf area calculation method was based on the previous research (Sun et al., 2006) then we made the modification according to the shape of the leaves we tested.

Totally 18 pair of plants were replicated to test, so we collected the data of *B. tabaci* adults number from 54 pairs of leaves. The density of eggs deposited by adults on corresponding leaves was also calculated 3 days post the infestation.

### *M. persicae* Feeding

Leaves of the plants treated using the method mentioned in section “Plant Materials, Insects, and Viruses” were harvested and cut into the pieces of 1.5 cm^2^ × 1.5 cm^2^. Then each piece of leaf was placed in a dish together with a moist piece of filter paper in order to keep the leaf fresh. Then one aphid nymph born within the last 5 h was placed on the leaf. The length of each nymph stage and adult stage, and the number of offspring was recorded by observation in a fixed time (3:00 p.m.) each day. The leaves for the feeding were changed every second days. Fourty bio-replicates were used. The longevity of each instar of nymphs and adults was recorded as well as the number of offspring laid by the adults.

### Symptoms Evaluation for TMV Assay

For the TMV assay a mechanical inoculation method was used. First we extracted the juice via grinding the virus-infected plant tissues contained TMV with 1 mL PBS buffer. Then we rubbed the same volume of juice we acquired on each inoculated leaves with emery. Finally we sprayed water on the inoculated leaves in order to wash away the emery. After the virus-inoculation, the average time before appearance of infection symptoms was recorded in order to figure out whether the herbicide residue can accelerate or slow down the progress of virus-infection. Consequently, disease severity was evaluated 5, 10, 15, and 20 days after the inoculation, according to Zhao et al. (2007) with the following modifications.

We divided the symptom into five degrees:

0 – no symptom;

1 – slight symptom including slight mosaic or slight deformation of no more than 1/3 of the leaves;

2 – 1/3 to 1/2 of leaves showed of mosaic or deformation;

3 – 1/2 to 2/3 of leaves had TMV correlated symptoms;

4 – more than 2/3 of leaves had TMV correlated symptoms.

Thirty bio-replicates were sued in this experiment. In addition to the above, all leaves were photographed for the records so that it is possible to do a re-evaluation and also make comparisons with other experiments.

### RNA Extraction and Reverse Transcription

A fixed amount of leaf samples (0.15 g) were harvested and ground in liquid nitrogen 10 days post the treatment of herbicide residue. Total RNA was extracted using the Tiangen plant RNA extraction prep kit (No. DP-432) following the manufacturers protocol and samples were collected in 1.5 mL Eppendorf tubes and kept at -80°C for the further analysis.

Total RNA was used for reverse transcription according to the instruction for the Promega reverse transcripts kit (No. A5001). The resulting cDNA used later as template was kept at -20°C.

### Determination of TMV Viral Replication

*Tobacco mosaic virus* inoculation was described in section “Symptoms Evaluation for TMV Assay,” while the method for total RNA extraction was given in section “RNA Extraction and Reverse Transcription.” We used RT-qPCR method was used to compare the amount of TMV in different treated plants. A SYBR Green kit (Promega, Product No. A6002) was used for this experiment. The 2^-ΔΔCt^ method was used to quantify the amount of TMV. *NtACTIN* (GenBank No. U60495.1) was used as housekeeping gene for data normalization. The TMV sequence was downloaded from NCBI (GenBank No. AF395127.1). Twelve biological replicates (each biological replicate includes three technical replicates) were performed for this assay.

### Endogenous JA and SA

Jasmonic acid and SA extraction and determination protocols has been described in earlier studies (Pan et al., 2010). High performance liquid chromatography and mass spectrometry (LC-MS) methods were used for the experiments. The conditions used for the analysis was described by Pan et al. (2010). This assay was completed at Institute of Genetics and Developmental Biology, Chinese Academy of Science.

The RT-qPCR method and the 2^-ΔΔCt^ were used to determine the expression of genes located in signal pathways (Turner et al., 2002; Shah, 2003; Chen et al., 2009; Katou et al., 2013). *NtWIPK* (Access No. D61377.1), *NtPDF1.2* (Access No. JQ654634.1), *NtLOX* (Access No. X84040.1), *NtPLD* (Access No. AF223573.1), *NtAOS* (Access No. AB778304.1), *NtAOC* (Access No. AJ308487.1), *NtOPR* (Access No. AB233416.1) were used for analysis to reflect the endogenous JA levels in plants exposed to BSM treatment. The housekeeping gene *NtActin* (Access No. U60495.1) in *N. tabacum* was used as internal reference to correct the result. *NtPR1a* (GenBank No. X12737.1), *NtPAL* (GenBank No. AB008199.1), *NtSABP* (GenBank No. AY485932.1), *NtICS1* (GenBank No. AY740529.1), and *NtSIPK* (GenBank No. U94192.1) was used to represent the endogenous SA response to soil BSM treatment. *NtACTIN* (GenBank No. U60495.1) and *NtG6PDH* (GenBank No. AJ001772.1) were used as housekeeping genes for data normalization. *NtRDR1* (GenBank No. XM_016610634.1) and *NtRDR6* (GenBank No. FJ966891.1) were studied as viral infection related genes.

### Data Analyses

In pest-infestation experiment, students’ *t*-tests were used to compare the density of adults on the leaves belonging to BSM treatment to controls at different time points. The same method was used to compare the egg-depositing by the adults, the symptom levels caused by TMV inoculation, TMV amount in plants, and the level of endogenous JA and SA as well as the expression of their responsive and biosynthetic genes. The statistical analysis was performed using of the statistical package SPSS19.0 (SPSS Inc., Chicago, IL, USA).

We used “Age-stage, Two-sex life” table method to evaluate the lifespan of *M. persicae* feeding on different types of leaves (Chi and Liu, 1985; Chi, 1988). The results included the longevity of each insect stage, the morality of nymphs, net reproductive rate (R_0_), gross reproduction rate (GRR), mean fecundity per female adult (F), intrinsic rate (r_m_), finite rate of population (λ), mean generation time (T), and longevity, the meaning of these results can be referred to the study performed by Huang and Chi (2013).

## Results

### BSM Residue Alters the Interaction between Host Plants and Whitefly *B. tabaci*

First, we tested the effects of different concentrations of BSM treatment of soil (2.0, 1.0, 0.25, 0.1, 0.05, and 0 mg⋅kg^-1^ as the control) on tobaccos for *B. tabaci* infestation and found that 0.25 mg⋅kg^-1^ is a suitable concentration for further experiments, as recommended in the fields applications. Higher concentrations could kill the plants, while the lower concentrations showed no noticable effect on susceptible plants for *B. tabaci* infestation.

**FIGURE 1:**
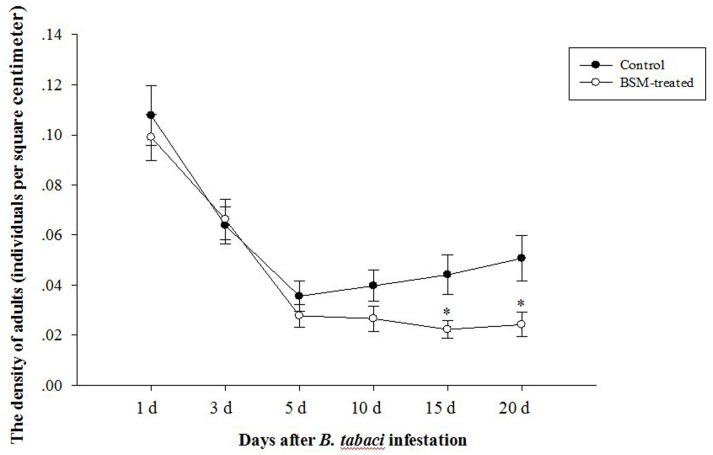
**Bensulfuron-methyl (BSM)-treated *Nicotiana tabacum* could attract less whitefly *Bemisia tabaci* but only in the later time post the infestation.** Tobaccos were used 10 days after treated with BSM in the soil, and the control type was set without chemical-treatment. Asterisks means significant difference occurred between two groups at the same time point (*n* = 54, ^∗^*p* < 0.05).

Bensulfuron-methyl-induced symptoms such as malformed leaves and chlorosis on treated plant tissue became apparently before the infestation of pests. Both of the densities of adult *B. tabaci* feeding on two different treated tobaccos (**Figure 1**) reduced quickly. The difference of densities of adult *B. tabaci* between control and treated plants were not significant during the early infection, but were increasingly apparent after 10 days, with *F* = 4.385, *df* = 106, *p* = 0.014 at 15 days and *F* = 6.361, *df* = 106, *p* = 0.013 at 20 days post-infestation, as BSM treatment reduced the infestation of *B. tabaci*.

We also counted the number of eggs on correlated leaves deposited by adult *B. tabaci* and determined the breeding preference of whitefly adults. But there was no significant difference in breeding preference with or without BSM-treatment according to the students’ *t*-test (*F* = 1.818, *df* = 106, *p* > 0.05).

### Effect of BSM on *M. persicae* Infestation of Plants

Aphids such as *M. persicae* fed on tobacco leaves similar to the whitefly experiments described above, but our result showed no significant difference in the longevity of each stage of *M. persicae* feeding on BSM-treated and control leaves, also feeding on BSM-treated plants had no remarkable impact on the morality of nymphs (**Table 1**).

**Table 1:** The longevity and the nymph-morality of *M. persicae* feeding on leaves from BSM-treated and control tobaccos.

Indexes	Control	BSM-treated (0.25 mg⋅kg^-1^)
The longevity of 1st nymph (day)	2.150 ± 0.170	2.080 ± 0.150
The longevity of 2nd nymph (day)	2.100 ± 0.130	1.900 ± 0.130
The longevity of 3rd nymph (day)	2.200 ± 0.230	1.840 ± 0.120
The longevity of 4th nymph (day)	1.710 ± 0.110	1.940 ± 0.160
The longevity of adult (day)	8.030 ± 0.620	9.500 ± 0.770
Morality of nymph	0.150 ± 0.056	0.100 ± 0.047


*In this table, fecundity value = 1, time unit = 1, *n* = 40. The data consists of mean value ± SE.*

Then we determined the longevity and the fecundity of the insects and we also found no significant difference between the groups feeding on BSM-treated and healthy plants (**Table 2**). All the results shown in the table were acquired using the method of Chi and Liu (1985) and Chi (1988).

**Table 2:** The lifetable of *M. persicae* feeding on leaves of BSM-treated and control tobaccos.

Indexes	Control	BSM-treated (0.25 mg⋅kg^-1^)
Intrinsic rate r_m_ (day)	0.232 ± 0.018	0.249 ± 0.016
Finite rate of population λ	1.261 ± 0.022	1.283 ± 0.021
Net reproductive rate R_0_	12.6 ± 2.003	15.925 ± 2.321
Mean generation time T (day)	10.920 ± 0.408	11.112 ± 0.526
The gross reproduction rate GRR	26.76 ± 5.218	30.46 ± 5.086
Mean fecundity per female adult F	14.82 ± 2.148	17.69 ± 2.407
Longevity (day)	14.8 ± 0.893	16.35 ± 1.028


*In this table, fecundity value = 1, time unit = 1, *n* = 40. The data consists of mean value ± SE.*

### BSM Treatment Reduce the Symptom of TMV Infection

**FIGURE 2:**
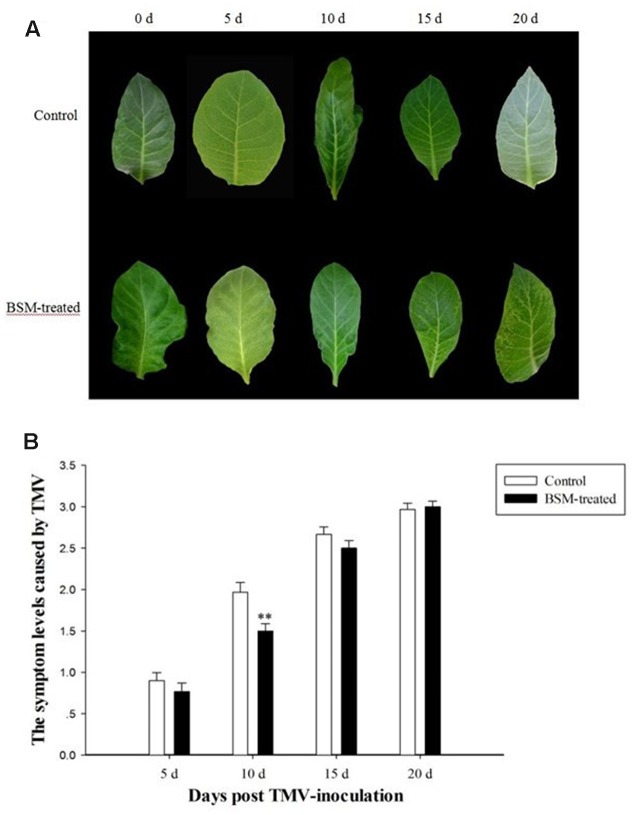
**The dynamic of symptom caused by *Tobacco mosaic virus* (TMV) on tobacco leaves treated with BSM in the soil.** Tobaccos were treated with BSM 10 days before TMV inoculation. **(A)** The symptom on the lobus cardiacus of *N. tabacum* in different time points post-TMV inoculation. Time points on the head of the figure indicated the time post-TMV inoculation. **(B)** The level of the symptom on leaves of BSM-treated and control tobaccos after the inoculation of TMV. Asterisks indicate that significant differences in each treated lines from control lines (*n* = 30, ^∗^*p* < 0.05, ^∗∗^*p* < 0.01).

*Tobacco mosaic virus* was inoculated on tobacco plants after 10 days of BSM treatment, and viral lesions appeared at about the same time as the control plants without BSM treatment, although the lesion was less severe as evidenced by lighter color (**Figure 2A**). But after the time point of 10 days post virus-inoculation, TMV-caused lesion on BSM-treated plants was not less severe any more. This result was in keeping with the evaluation of the symptom levels. BSM-treated plants also had lower level of virus-symptoms compared to the plants not treated by herbicide. As the development of the virus-induced symptom, and the accumulation of the virus, there was no significant difference in the viral lesions and symptoms if TMV was inoculated 15 or 20 days after BSM treatment (**Figure 2B**).

### BSM Treatment Reduces Early RNA Replication

**FIGURE 3:**
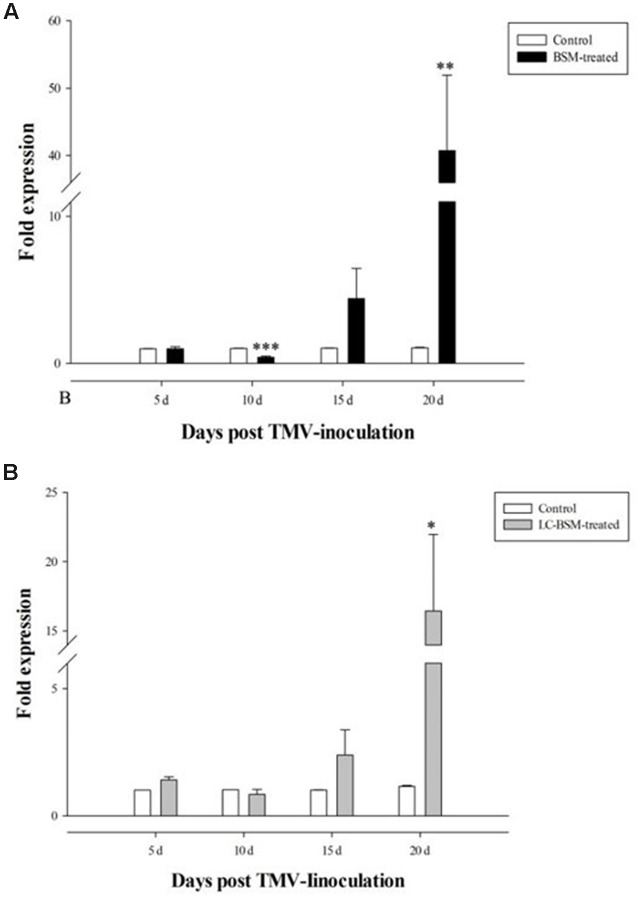
**The expression of TMV coat protein gene in *N. tabacum* in different time points post-inoculation.** Tobaccos for the inoculation were treated by different concentration of BSM for 10 days. 2^-ΔΔCt^ method was applied in the experiment. **(A)** The expression of TMV gene in both BSM-treated and control lines, and asterisks indicate that significant differences in each treated lines from control lines (*n* = 12, ^∗^*p* < 0.05, ^∗∗^*p* < 0.01, ^∗∗∗^*p* < 0.001). **(B)** LC-BSM indicated low-concentration of BSM applied in the experiment. Asterisks indicate that significant differences in each treated lines from control lines (*n* = 12, ^∗^*p* < 0.05, ^∗∗^*p* < 0.01, ^∗∗∗^*p* < 0.001).

Next we determined the effect of BSM treatment on TMV RNA replication upon infection of the susceptible tobacco strain K326, in comparison to control plants without BSM treatment. In this regard, we used RT-qPCR to determine the level of coat protein (CP) to reflect virus replication in these tobacco plants, and found that early viral replication was significantly lower in BSM-treated tobacco leaves (**Figure 3A**). Interestingly, the situation changed during later viral infection, with dramatic increase of viral RNA replication in BSM-treated plants at 15- and 20-day post-infection, indicating that BSM treatment only delayed viral RNA replication rather than directly blocking the process *per se*.

Since our result showed that BSM residue in the soil strongly affected viral RNA replication in the plants, we wondered if lower BSM concentrations were also effective. To this end, we performed the same TMV experiments as described above except with fivefold lower BSM concentration (0.05 mg⋅kg^-1^). Our result showed no significant difference in early viral replication (10 days post-infection) with or without BSM treatment (*F* = 24.413, *df* = 22, *p* > 0.05), while this low BSM concentration remained effective in boosting late viral RNA replication (20 days post-infection) albeit to a lesser extent than higher BSM concentrations (**Figure 3B**).

### BSM Treatment Induces JA and SA Production, As Well As the Expression of Responsive and Biosynthetic Genes

**FIGURE 4:**
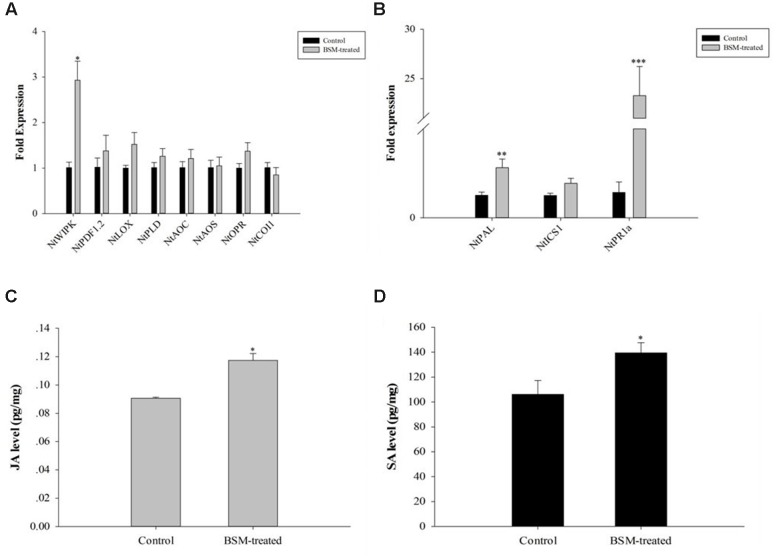
**jasmonic acid (JA) and salicylic acid (SA) level in *N. tabacum* was induced by BSM treatment in the soil as well as the key genes related to response and biosynthesis.** Tobaccos were treated with BSM in the soil 10 days prior to the phytohormones and the expression of associated genes determination. **(A)** The expression of JA marker genes in treated and control lines, 2^-ΔΔCt^ method was applied in the experiment. Asterisk indicates significant difference from the control type (*n* = 3, ^∗^*p* < 0.05); **(B)** The expression of SA marker genes in treated and control lines, 2^-ΔΔCt^ method was applied in the experiment. Asterisk indicates significant difference from the control type (*n* = 3, ^∗^*p* < 0.05, ^∗∗^*p* < 0.01, ^∗∗∗^*p* < 0.001); **(C)** The level of JA in treated and control groups. Asterisk indicates significant difference from the control type (*n* = 3, ^∗^*p* < 0.05); **(D)** The level of SA in treated and control groups. Asterisk indicates significant difference from the control type (*n* = 3, ^∗^*p* < 0.05).

Jasmonic acid and SA signal transduction pathways are important for plant immune system to response to pathogen infections. Among the responsive and biosynthetic genes, we found that BSM treatment up-regulated only *NtWIPK* gene in the JA pathway (*F* = 12.558, *df* = 4, *p* = 0.024; **Figure 4A**), but up-regulated the expression of two genes, *NtPAL* (*F* = 3.910, *df* = 4, *p* = 0.009) and *NtPR1a* (*F* = 4.375, *df* = 4, *p* < 0.001), in the SA pathway (**Figure 4B**).

We determined the levels of JA and SA in BSM-treated and control plants. JA showed strong up-regulation (*F* = 4.649, *df* = 4, *p* = 0.029) 10 days after the BSM treatment of the soil (**Figure 4C**), consistent with the RT-qPCR results described above. In addition, BSM treatment also up-regulated the SA level in these plants (*F* = 0.493, *df* = 4, *p* = 0.014) also showed the same result as JA level in those plants (**Figure 4D**), indicating that both of the phytohormones were strongly induced by the herbicide-treatment.

### TMV Infection Induces Gene Expression Involved in SA Response and Biosynthesis

**FIGURE 5:**
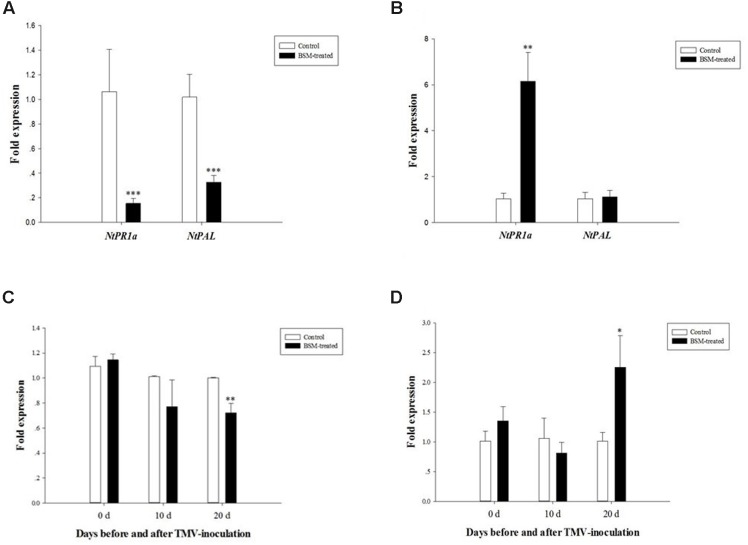
**The expression of *NtPR1a* and *NtPAL* in plants 10 and 20 days post-TMV inoculation, as well as the expression of *NtRDR1* and *NtRDR6* involved in virus-resistance 0, 10, and 20 days post virus-inoculation.** Plants first were treated with BSM in the soil for 10 days then used for virus-inoculation. **(A)** The expression of *NtPR1a* and *NtPAL* in BSM-treated and control lines 10 days post-inoculation, 2^-ΔΔCt^ method was applied in the experiment. Asterisk indicates significant difference from the control type (*n* = 3, ^∗^*p* < 0.05, ^∗∗^*p* < 0.01, ^∗∗∗^*p* < 0.001); **(B)** The expression of *NtPR1a* and *NtPAL* in BSM-treated and control lines 20 days post-inoculation, 2^-ΔΔCt^ method was applied in the experiment. Asterisk indicates significant difference from the control type (*n* = 3, ^∗^*p* < 0.05, ^∗∗^*p* < 0.01, ^∗∗∗^*p* < 0.001); **(C)** The expression of *NtRDR1* in both BSM-treated and control lines at different time points post-TMV inoculation. Asterisk indicates significant difference from the control type (*n* = 3, ^∗∗^*p* < 0.01). **(D)** The expression of *NtRDR6* in both BSM-treated and control lines at different time points post-TMV inoculation. Asterisk indicates significant difference from the control type (*n* = 3, ^∗^*p* < 0.05).

It was remarkable that the BSM-treated plants increased TMV replication during the later viral infection. In this regard, SA as well as related genes was significantly up-regulated in BSM-treated plants compared to the controls. Both PR1a and PAL play a key role in endogenous SA accumulation (Shah, 2003; Chen et al., 2009). Thus, we determined the expression of *NtPR1a* and *NtPAL* at 10- and 20-day post-inoculation, and found that both *NtPR1a* (*F* = 4.278, *df* = 4, *p* < 0.001) and *NtPAL* (*F* = 5.540, *df* = 4, *p* < 0.001) was down regulated in BSM-treated tobaccos 10-day post-TMV inoculation (**Figure 5A**), while *NtPR1a* (*F* = 13.854, *df* = 4, *p* = 0.007) expression recovered better in BSM-treated plants at 20-day post-TMV inoculation than in control plants (**Figure 5B**). The expression of *NtPAL* was not significantly different between treated and control plants at 20-day post-inoculation. Our result suggested that the SA level was suppressed by the TMV accumulation during early infection in BSM treated plants, which could contribute to the higher levels of TMV replication during late infection.

Since, RDR family of RNA polymerase (RDR) can also be regulated by endogenous SA (Lee et al., 2016). RDR1-6 contributes to antiviral silencing and symptom-limiting in plants (Lee et al., 2016), we determined the expression of *NtRDR1* and *NtRDR6* before the inoculation of TMV. However, the expression of both *NtRDR1* (*F* = 4.408, *df* = 4, *p* = 0.163) and *NtRDR6* (*F* = 1.194, *df* = 4, *p* = 0.379) didn’t show significant change (*F* = 4.408, *df* = 4, *p* = 0.163) upon BSM treatment (**Figures 5C,D**). Though both of RDR1 and RDR6 play substantial roles to assist plants to fight against virus, our data suggests that they are not involved the BSM-mediated response to TMV infection. We also determined the expression of *NtRDR1* and *NtRDR6* after the inoculation in both BSM-treated and control plants. At 20-day post-inoculation when there was high levels of TMV replication, *NtRDR1* showed remarkable down regulation (*F* = 4.478, *df* = 4, *p* = 0.003) while the expression of *NtRDR6* was significantly induced (*F* = 12.282, *df* = 4, *p* = 0.036).

## Discussion

Herbicide residue draws much more attention today than ever before because pose threaten to the crop yield and the safety of not only the environment but also the humanity (Nicosia et al., 1991). And great effort was made to solve the problem such as the detection of the residue and the biodegradation in the soil (Lin et al., 2012; Yang et al., 2012; Feng et al., 2013), or the target site in the target weeds in order to reduce the usage (Wei et al., 2015). An attempt was made to discover the interaction between crops and important pests/viruses under the residue of herbicide in the soil in our investigation.

We found that the residue of the herbicide could only cause the avoidance to *B. tabaci* of plants during the later infestation. The performance of the wingless insect *M. persicae*, as well as the fecundity of both insects were not affected by the herbicide. However, the residue of BSM can strongly affect the activity of virus in plants. Thus we discover that JA and SA, which play significant roles in biotic interactions in plants, were both induced under the residue of the herbicide in the soil. We also found that SA pathway could be inhibited under the simultaneous existence of TMV and herbicide. We also focus on the factors such as RDRs related to the virus-resistance in plants and found that they could only affected by the virus, but not the herbicide. In this study, the results we found needed to be discussed.

There has been few investigations focusing on the interaction between plants and insects under the application of other herbicides such as atrazine or other kinds of sulfonylurea herbicides (Dewey, 1986; Kjaer and Heimbach, 2001). In the study of atrazine in 1986, the researchers focused on the community of the insects and suggested that herbicides affected the community of the insects community indirectly by reducing the food and habitat (Dewey, 1986). In the study of sulfonylurea herbicide application on different host plants which were not sensitive, the activity of insects feeding on them were not severely affected except the death rate of *Gastrophysa polygoni* larvae (Kjaer and Heimbach, 2001). In our investigation, first of all, tobaccos are sensitive to BSM residues and show remarkable symptoms. In the second place, we study the interactions between the plants and insects from the aspect of physiology including the changing of JA and SA production. According to the fecundity determination assay, in some investigations, the activity of the pests can increase the production of SA (Shi et al., 2014b; Cao et al., 2016), while SA in some cases can also inhibit the activity of pests (Rodriguez-Alvarez et al., 2015). Although in our investigation, the fecundity of both *M. persicae* and *B. tabaci* was not significantly affected, this was in correlation with the study performed by Kjaer and Heimbach (2001). This also indicates the limitation of herbicide on the activity of insects, because the target are in the weeds and not in the insects.

Similarly, few studies have paid attention to the interaction between plants and pathogens as a consequence of herbicide-treatment of. We have found no study focussing on the effect of sulfonylurea herbicide for this interaction. The speculative effects of herbicide application on the invasion of viruses can be divided into the following: plants were not affected by herbicide; plants were affected by the herbicide, the virus concentration was suppressed, enhanced or not affected (Kazinczi et al., 2003). Previous investigations has generally showed that the activity of viruses are inhibited by the application of herbicides (Kazinczi et al., 2006; Hooks et al., 2009). Krcatovic et al. (2008) point out that the treatment of some metabolites such as flavonoids, quercetin, and vitexin on plants also can only suppress the activity of TMV during early infection accompanied with the induce of SA, but the phenomenon cannot last for a long time. This was only consistent with the early infection of TMV in tobaccos treated with BSM in our study. Furthermore, the susceptibility of BSM-treated tobaccos to TMV during the later infection was first shown in the present investigation.

In many studies performed before, JA and SA pathways are presented as antagonists (Turner et al., 2002; Lyons et al., 2013). The suppression of target genes related to JA can lead to the accumulation of SA (Turner et al., 2002). In some studies focused on the plant-insect interactions, the infestation of pests can lead to the suppression of JA level and the enhancement of SA production (Shi et al., 2014b). Some investigations focusing on the relationship between herbicides and SA pointed out that SA and H_2_O_2_ shared a common signaling pathway, this indicated that SA in rapeseed could be boosted by the treatment of other herbicide such as napropamide (Cui et al., 2010). According to the results of the TMV assay above, the concentration of virus is lower in BSM-treated plants during early infection, this correlates with the result that TMV can be inhibited by the exogenous treatment of SA (Conti et al., 2012). However, the fact that not only increased SA level in sensitive plants exposed to the treatment of sulfonylurea herbicide, but the JA production was affected by the application of herbicide was first discovered in our study, and the result that both of them showed up-regulation are different to previous studies.

In the further investigation, higher expression of genes in SA signal pathway was inhibited by lower concentration of TMV in BSM-treated plants. Thus, we speculate the inhibited SA signal pathway can lead to the higher viral replication. And in this regard, SA production in BSM-treated tobaccos can be more easily blocked in the presence of both virus-inoculation and herbicide-treatment. In this assay, we didn’t take JA into consideration because the role of JA involved in the anti-virus is still unclear. Oka et al. (2013) discovered the negative impact of JA on virus-resistance in plants because they found that the application JA in tobaccos (Shannon variety, which is resistant to TMV) can lead to the lesion on leaves. Zhu et al. (2014) performed the exogenous JA and JA-defective mutants assay and discovered that without JA, the plants could be more sensitive to viruses. But the mechanism that SA biosynthesis can be suppressed in plants exposed to the presence of both virus and BSM needs to be further investigated.

*RNA-dependent RNA polymerase* family though plays a key role in resistance to the infection by the virus. It can mediate the conversion of viral single-strand RNA to double-strand RNA, then lead to further degradation (Harmoko et al., 2013). The function of RDR1 and RDR6 was studied most. But those studies mainly focused on virus-resistance (Di Serio et al., 2010; Jiang et al., 2012). For example, Rodriguez et al. (2014) found that RDR1 located in the downstream of SA signal could be inhibited by the infection of TMV. In addition, RDR6 in rice plays key roles in fighting against not only viruses, but also fungus and bacteria (Wagh et al., 2016). The interaction between RDR family and herbicide was not reported in any study. In our investigation, the activity of RDR family cannot be affected by the residue of herbicide in the soil, though the signal pathway of SA, as well as the infection of virus were affected by the herbicide.

## Conclusion

The study investigated the performance of the pests and viruses in susceptibility plants *N. tabacum* treated with BSM soil residues. The results pointed out that plants can be more resistant to *B. tabaci* only during the laer time post-infestation in treatment when BSM residues are present in the soil. However, the longevity of both development stage and life of *M. persicae* was not affected, nor was the fecundity of both insects. Although plants treated with BSM could be more resistant to TMV accumulation during the early infection, the viral RNA replication was enhanced during the late infection in these plants, and this phenomenon was weaker at lower concentration of BSM. The expression of marker genes related to JA and SA was up-regulated, as well as the contents of both phytohormones. Although, during the early infection of TMV in BSM-treated plants, the expression of SA targeted genes was strongly inhibited, thus we speculate that this was beneficial for the multiplication of TMV. In addition, both of *NtRDR1* and *NtRDR6* were not involved in the anti-virus which was affected by the application of BSM in our study. This study revealed the effect brought by the residue of herbicide in the soil on fighting against pests and viruses of plants. The correlated physiological phenomenon was also displayed. The study can contribute to the study on the ecosystem of cropland suffering the pollution of the herbicide.

## Author Contributions

RL completed the whole research work, summarized the correlated data, and composed the draft. SUI contributed to the assistance of research work and the English correction of the writing. ZW and XY provided the guidance of the work and made some suggestions about the work.

## Conflict of Interest Statement

The authors declare that the research was conducted in the absence of any commercial or financial relationships that could be construed as a potential conflict of interest.

The reviewer JV and handling Editor declared their shared affiliation, and the handling Editor states that the process nevertheless met the standards of a fair and objective review. The reviewer ZW declared a shared affiliation, though no other collaboration, with the authors to the handling Editor, who ensured that the process nevertheless met the standards of a fair and objective review.
